# Generalization of the Model of Magnetoelastic Effect: 3D Mechanical Stress Dependence of Magnetic Permeability Tensor in Soft Magnetic Materials

**DOI:** 10.3390/ma13184070

**Published:** 2020-09-14

**Authors:** Roman Szewczyk

**Affiliations:** Warsaw University of Technology, Faculty of Mechatronics, Institute of Metrology and Biomedical Engineering, A. Boboli 8, 02-525 Warsaw, Poland; roman.szewczyk@pw.edu.pl; Tel.: +49-609-464741

**Keywords:** magnetic permeability tensor, magnetoelastic effect, principal stresses

## Abstract

This paper presents a new solution enabling modeling of the mechanical stress tensor dependence of the 3D relative permeability tensor of isotropic material only on the basis of knowledge of the axial stress dependence characteristics. For the proposed model, the concept of principal stresses is utilized. In such a case, the sophisticated system of axial and shear stresses may be reduced to the set of axial stresses in a rotated coordination axes system. As a result, the proposed solution generalizes the explanation of the shape of magnetoelastic characteristics as well as radically extending possibility of the application of the finite elements methods (FEM) to describe sophisticated magnetoelastic systems.

## 1. Introduction

The stress dependence of the magnetic permeability of soft magnetic materials is the subject of intensive investigation [1,2]. However, these experimental investigations are focused on the simplified, preferably uniform distribution of axial [3,4] or shear [5] stresses. The presented results of experimental measurements clearly indicated that the influence of stress has great technical importance due to significant changes in the magnetic permeability of soft magnetic materials subjected to stress from external forces [6]. These changes in magnetic permeability can exceed 50% in newly developed magnetic materials, such as soft ferrites [7] or amorphous [8] and nanocrystalline materials [9].

For this reason, the mechanical stress dependence of the functional parameters of components made of soft magnetic materials, such as magnetic cores or other elements, have to be modeled in order to ensure an efficient application process [10,11]. On the other hand, mechanical stress distribution in technical components is often sophisticated. In addition, axial and shear stresses occur at the same time. Until now, it was not possible to connect the results of measurements of uniform stress dependence characteristics with the stress dependence of magnetic characteristics of real technical objects.

The presented paper fills this gap. The model describing the influence of a mechanical stress tensor on the magnetic permeability tensor of isotropic materials is proposed. To determine the influence of both axial and shear stresses acting simultaneously, the concept of principal stresses is used. As a result, the magnetic permeability tensor of the magnetic material subjected to sophisticated stresses can be efficiently calculated. This opens up new possibilities for the finite elements method based on modeling the stress dependence of the magnetic characteristics of inductive components utilized in real applications.

The generalization of the model of magnetoelastic effect focused on the influence of a 3D stress tensor on the magnetic permeability tensor will enable optimization of bioengineering sensors [12,13] used for in vivo skin curvature measurements. Moreover, quantitative explanation of the principles of operation and optimization of the geometry of torque sensors [14] will be possible. Finally, it should be highlighted that the principles of operation of large industrial magnetomechanical sensors—pressductors [15] or accurate tensductors [16]—are connected with mechanical stress-induced changes in the permeability tensor, not changes in the sensor’s dimensions. As a result, on the basis of the presented generalization, the operation of pressductors and tensductors may be quantitatively described. This will lead to optimization of these sensors, increases in their sensitivity and size reduction.

## 2. Model of the Magnetoelastic Effect

Mechanical stresses cause changes in the total free energy of a magnetic material [17]. This change is connected with the appearance of magnetoelastic energy *E_σ_* given as Equation (1) [18]

(1) $$E_{\sigma }=\frac{3}{2}\lambda_{s}\sigma$$

where *λ_s_* is saturation magnetostriction, whereas *σ* is the value of axial stresses. In the case of isotropic material, additional magnetoelastic energy *E_σ_* causes axial anisotropy. However, it should be considered that *λ*_s_ is also stress-dependent [19], which should be considered for higher values of mechanical stress. In addition, for analyses of magnetoelastic energy *E_σ_*, the anisotropy of the magnetic material as well as the boundary conditions should be considered.

The influence of mechanical stresses on the anhysteretic magnetization curve *M*(*H*) (neglecting magnetic hysteresis [16]) may be estimated on the basis of the following Equations (2)–(4) [20,21]:

(2) $$M\left(H\right)=M_{s}\left(\frac{\int_{0}^{\pi }e^{\frac{E\left(1\right)+E\left(2\right)}{2}}\cdot \sin\theta \cdot cos\theta \cdot d\theta }{\int_{0}^{\pi }e^{\frac{E\left(1\right)+E\left(2\right)}{2}}\cdot \sin\theta \cdot d\theta }\right)$$

(3) $$E\left(1\right)=\frac{H_{e}}{a}cos\theta -\frac{E_{\sigma }}{M_{s}\cdot \mu_{0}\cdot a}sin^{2}\left(\psi -\theta \right)$$

(4) $$E\left(2\right)=\frac{H_{e}}{a}cos\theta -\frac{E_{\sigma }}{M_{s}\cdot \mu_{0}\cdot a}sin^{2}\left(\psi +\theta \right)$$

where *M_s_* is saturation magnetization, *μ_0_* is the magnetic constant, ψ is the angle between the magnetizing field *H_e_* and the direction of axial stresses. Similarly to the Jiles–Atherton model of magnetic hysteresis [22], effective magnetic field *H_e_* is given as Equation (5) [23]

(5) $$H_{e}=H+\alpha M$$

where *H* is the external magnetizing field and *α* is Bloch’s interdomain coupling. Moreover, according to the Jiles–Atherton model, the parameter *a* describes the domain wall density and is defined as Equation (6) [23]

(6) $$a=\frac{N\cdot k_{B}\cdot T}{\mu_{0}\cdot M_{s}}$$

where *N* is magnetic domain density, *k_B_* is Boltzmann’s constant and *T* is temperature. Finally, the relative magnetic permeability *μ(H)* can be easily calculated as Equation (7):

(7) $$\mu \left(H\right)=\;\frac{M\left(H\right)}{H}$$

where *M(H)* is the magnetization of the material given in (A/m). Due to the fact that the integrals in Equation (2) have no antiderivatives, numerical analyses indicate that the axial stress *σ* dependence of relative magnetic permeability m can be described by linear approximation Equation (8):

(8) $$\mu \left(\sigma \right)=\mu_{u}+k\cdot \sigma$$

where *μ_u_* is the relative magnetic permeability for the unstressed sample and *k* is the axial stress sensitivity of the magnetic material. It should be highlighted that parameter *k* may be positive or negative according to the positive or negative value of the material’s saturation magnetostriction *λ_s_* [7]. Linear approximation given by Equation (8) is valid for small values of both tensile and compressive stresses *σ* (where saturation magnetostriction *λ_s_* may be considered as constant [19]) as well as for the initial part of the magnetization curve, where relative magnetic permeability may be approximated as a constant value. It should be highlighted that, for the case of uniform axial stresses, *μ(σ)* characteristics are widely presented in the literature for different magnetic materials [24,25].

In technical systems, mechanical stresses are often not parallel to the direction of the magnetizing field. However, results of Barkhausen noise analysis confirmed [26] that effective axial stresses *σ_e_* influencing the magnetic properties of magnetic materials may be calculated from the following Equation (9) [27]:

(9) $$\sigma_{e}=\;\sigma \cdot \left(sin\varphi -\nu \cdot cos\varphi \right)$$

where *σ* represents mechanical stresses from external forces, *ν* is the Poisson’s ration and *φ* is the angle between the direction of the magnetizing field and the direction of mechanical stresses. It can be easily calculated that, for mechanical stresses perpendicular to the magnetizing field, effective stresses *σ_e_* influencing the magnetic properties are given as Equation (10):

(10) $$\sigma_{e}=\;-\nu \cdot \sigma$$

## 3. Proposed Description of the Mechanical Stress Dependence of 3D Magnetic Permeability Tensor

In the case of isotropic magnetic material, its relative magnetic permeability ***μ*** may be presented as the tensor Equation (11):

(11) $$\mu =\left[\begin{array}{ccc} \mu_{xu} & 0 & 0\\ 0 & \mu_{yu} & 0\\ 0 & 0 & \mu_{zu} \end{array}\right]$$

where *μ_xu_* = *μ_yu_* = *μ_zu_* = *μ_u_* is the relative magnetic permeability of isotropic material without stresses.

In the case of anisotropic materials, such as electrical steels, both stress-induced anisotropy and magnetocrystalline anisotropy occur in the total free energy balance of the material. However, the interaction between stress-induced anisotropy energy and magnetocrystalline anisotropy energy is not clear. Understanding of this interaction requires both experimental investigation as well as micromagnetic analysis considering relative physics dependences. For this reason, the analyses presented in the paper are limited only to isotropic materials, where only stress-induced anisotropy occurs.

For isotropic magnetic material subjected to axial mechanical stresses *σ_xx_, σ_yy_* and *σ_zz_* acting along the 3D axes’ directions, considering the Equations (8) and (10), the stress dependence of the relative magnetic permeability tensor components is given as Equations (12)–(14):

(12) $$\mu_{xx}\left(\sigma_{xx},\sigma_{yy},\sigma_{zz}\right)=\mu_{u}+k\cdot \sigma_{xx}-\nu \cdot k\cdot \sigma_{yy}-\nu \cdot k\cdot \sigma_{zz}$$

(13) $$\mu_{yy}\left(\sigma_{xx},\sigma_{yy},\sigma_{zz}\right)=\mu_{u}+k\cdot \sigma_{yy}-\nu \cdot k\cdot \sigma_{xx}-\nu \cdot k\cdot \sigma_{zz}$$

(14) $$\mu_{zz}\left(\sigma_{xx},\sigma_{yy},\sigma_{zz}\right)=\mu_{u}+k\cdot \sigma_{zz}-\nu \cdot k\cdot \sigma_{xx}-\nu \cdot k\cdot \sigma_{yy}$$

**Figure 1:**
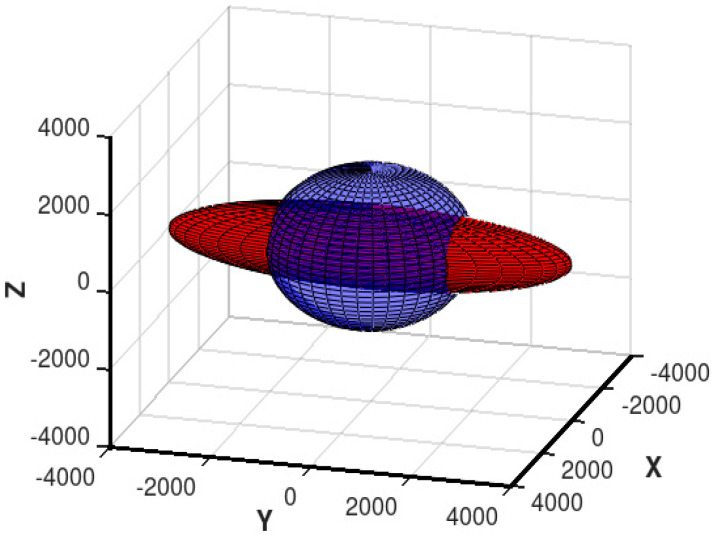
The stress dependence of relative magnetic permeability tensor ***μ*** of isotropic material: blue sphere—relative permeability tensor of unstressed material, red ellipsoid: relative permeability tensor of magnetic material subjected to tensile stresses in the *Y*-axis direction (positive value of *k* parameter).

The example of the influence of mechanical stresses applied to the isotropic material in the *Y*-axis direction on its relative magnetic permeability tensor ***μ*** is presented in Figure 1. As it can be observed, for a positive value of *k* parameter and tensile stresses *σ_yy_*, relative magnetic permeability may be presented as an ellipsoid with the major axis corresponding to the mechanical stress *Y*-axis direction.

In the case of a real system, where both axial *σ* and shear stresses *τ* occur, mechanical stresses are described by the ***σ*** tensor Equation (15):

(15) $$\sigma =\;\left[\begin{array}{ccc} \sigma_{xx} & \tau_{xy} & \tau_{xz}\\ \tau_{yx} & \sigma_{yy} & \tau_{yz}\\ \tau_{zx} & \tau_{zy} & \sigma_{zz} \end{array}\right]$$

**Figure 2:**
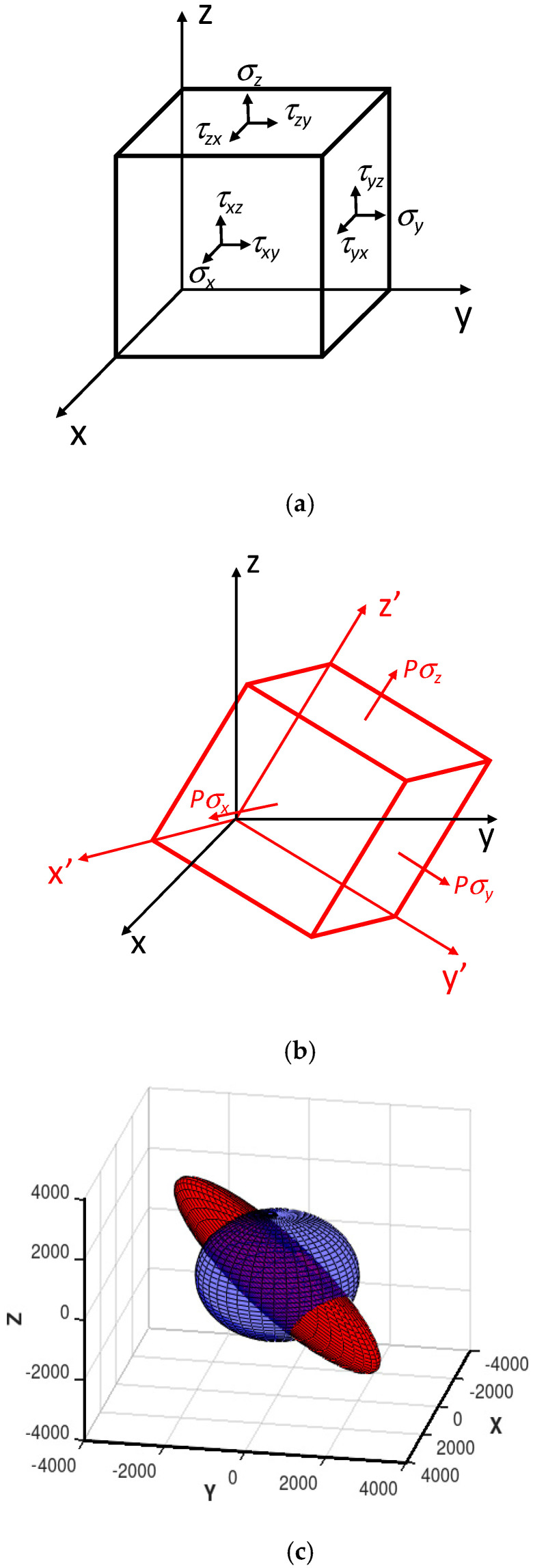
The idea of the method of estimation of stress tensor ***σ*** dependence of relative magnetic permeability ***μ*** tensor: (**a**) visual presentation of mechanical stress tensor ***σ*** components; (**b**) principal stresses ***Pσ_X’Y’Z_*_’_** in rotated coordinated axes system; (**c**) relative permeability tensor of unstressed material (blue sphere), relative permeability tensor ***μ*** of magnetic material subjected to stress tensor ***σ*** represented by principal stress tensor ***Pσ_X’Y’Z’_*** in rotated coordinating system X’Y’Z’ (red ellipsoid).

Graphical representation of stress tensor ***σ*** is given in Figure 2a. Unfortunately, there has been no physical model presented previously which enables consideration of the influence of both axial *σ* and shear stresses *τ* simultaneously. However, as it was proven previously [28], each stress tensor ***σ*** can be reduced to axial stresses tensor ***σ’*** in the rotated coordinate system X’Y’Z’. Such rotations are presented in Figure 2b. Directions of X’Y’Z’ are called principal directions, whereas axial stresses in such a system are known as principal stresses *Pσ_X’_*, *Pσ_Y’_* and *Pσ_Z’_* [29,30], forming a diagonal tensor Equation (16):

(16) $$P\sigma_{X^{\prime }Y^{\prime }Z^{\prime }}=\;\left[\begin{array}{ccc} P\sigma_{X^{\prime }} & 0 & 0\\ 0 & P\sigma_{Y^{\prime }} & 0\\ 0 & 0 & P\sigma_{Z^{\prime }} \end{array}\right]$$

Coordinate systems XYZ and X’Y’Z’ are connected by the so-called rotation matrix ***R***, given as Equation (17) [31]:

(17) $$R=\left[\begin{array}{ccc} \cos\left(X^{\prime },X\right) & \cos\left(X^{\prime },Y\right) & \cos\left(X^{\prime },Z\right)\\ \cos\left(Y^{\prime },X\right) & \cos\left(Y^{\prime },Y\right) & \cos\left(Y^{\prime },X\right)\\ \cos\left(Z^{\prime },X\right) & \cos\left(Z^{\prime },Y\right) & \cos\left(Z^{\prime },Z\right) \end{array}\right]$$

determined by the set of cosinuses of angles between the rotated X’Y’Z’ and original XYZ coordinate systems.

To calculate the relative magnetic permeability tensor ***μ*** influenced by stress tensor ***σ***, the permeability tensor’s dependence on principal stresses ***Pσ*** should be determined according to Equations (11)–(14):

(18) $$\mu \left(P\sigma_{X^{\prime }Y^{\prime }Z^{\prime }}\right)=\;\left[\begin{array}{ccc} \mu {\left(P\sigma \right)}_{X^{\prime }} & 0 & 0\\ 0 & \mu {\left(P\sigma \right)}_{Y^{\prime }} & 0\\ 0 & 0 & \mu {\left(P\sigma \right)}_{Z^{\prime }} \end{array}\right]$$

(19) $$\mu {\left(P\sigma \right)}_{X^{\prime }}=\;\mu_{u}+k\cdot P\sigma_{X^{\prime }}-\nu \cdot k\cdot P\sigma_{Y^{\prime }}-\nu \cdot k\cdot P\sigma_{Z^{\prime }}$$

(20) $$\mu {\left(P\sigma \right)}_{Y^{\prime }}=\;\mu_{u}+k\cdot P\sigma_{Y^{\prime }}-\nu \cdot k\cdot P\sigma_{X^{\prime }}-\nu \cdot k\cdot P\sigma_{Z^{\prime }}$$

(21) $$\mu {\left(P\sigma \right)}_{Z^{\prime }}=\;\mu_{u}+k\cdot P\sigma_{Z^{\prime }}-\nu \cdot k\cdot P\sigma_{X^{\prime }}-\nu \cdot k\cdot P\sigma_{Y^{\prime }}$$

Finally, the relative permeability tensor ***μ(σ)*** dependent on principal stresses ***Pσ_X’Y’Z’_*** calculated in Equations (19)–(21), should be rotated by rotation matrix ***R*** Equation (22), as presented in Figure 2c:

(22) $$\mu \left(\sigma \right)=R\cdot \;\mu \left(P\sigma_{X^{\prime }Y^{\prime }Z^{\prime }}\right)\cdot R^{T}$$

It should be highlighted that, as a result, it is possible to calculate the stress tensor dependence of the relative permeability tensor of isotropic material only on the basis of knowledge of the axial stress dependence characteristic.

## 4. The Possibilities of Practical Use of Proposed Generalization

**Figure 3:**
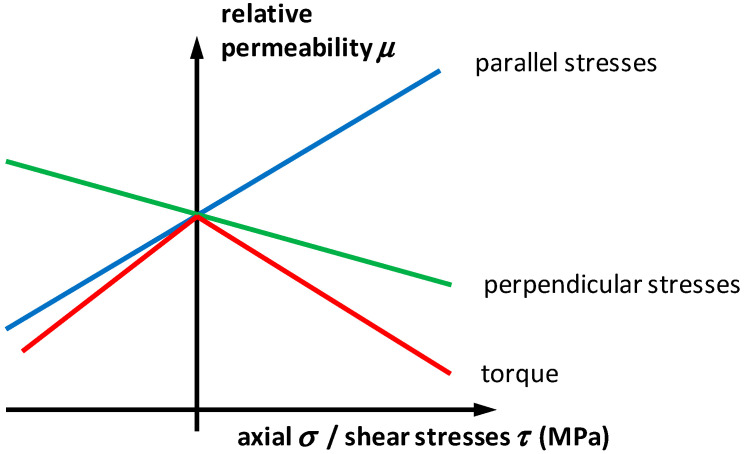
Generalization of the mechanical stress dependence of relative permeability of magnetic material with positive saturation magnetostriction *λ*_s_: blue—axial stresses parallel to the direction of magnetization [32], green—axial stresses perpendicular to the direction of magnetization [33], red—shear stresses dependence caused by the torque [35].

The proposed general description of stress tensor ***σ*** dependence of relative permeability tensor ***μ*** enables explanation of all magnetoelastic characteristics of isotropic or nearly isotropic soft magnetic materials. There are three most important cases: axial stress dependence with stresses parallel to the magnetizing field [32], axial stress dependence with stresses perpendicular to the magnetizing field [33] as well as shear stress dependence [34] often caused by torque [35]. The general explanation of the stress dependence of relative magnetic permeability is presented in Figure 3. In the case of magnetic material with positive magnetostriction *λ_s_*, for tensile axial stresses considered as positive, the value of relative permeability ***μ*** increases due to the fact that the major axis of the relative permeability tensor ellipsoid is aligned in the direction of the mechanical stresses. In the same case, for axial tensile stresses perpendicular to the magnetizing field, the value of relative permeability ***μ*** decreases due to the fact that the major axis of the relative permeability tensor ellipsoid is aligned perpendicularly to the mechanical stresses. In the third case, for a torque moment generating shear stresses *τ*, the relative magnetic permeability decreases independently in the shear stress direction. This phenomenon is caused by the fact that, in the case of shear stresses, principal stresses are created in the coordinate axes system rotated by 45° in the magnetizing field direction. As a result, shear stresses cause the decrease of magnetic permeability independently of its direction. This phenomenon is in very good agreement with experimental results presented previously in the literature [34,35].

In addition, the proposed solutions extend the possibility of the application of finite elements methods (FEM) to describe sophisticated 3D magnetoelastic systems. When solving the finite elements models of mechanical systems, both principal stresses ***Pσ_X’Y’Z’_*** as well as rotation matrix ***R*** are calculated on the basis of iterative optimization [36]. Moreover, the axial stress dependence of relative magnetic permeability can be estimated with the use of frame-shaped cores [37] or during the application of tensile force to the strips of material [38]. On the other hand, the proposed solution is limited to isotropic or nearly-isotropic materials such as soft ferrites, non-grain oriented electrical steels or amorphous alloys annealed without stresses or external magnetic fields.

## 5. Conclusions

The solution proposed in this paper overcomes the problem of the simultaneous presence of axial and shear stress in magnetoelastic systems with isotropic magnetic materials. On the basis of the principal stress tensor concept, the proposed solution enables modeling of sophisticated magnetomechanical systems only on the basis of knowledge about the axial stress dependence of the magnetoelastic characteristics.

As a result, the proposed method appears as a breakthrough concept for finite elements models of magnetomechanical systems. With the proposed procedure, bioengineering sensors [12,13], torque sensors [14] as well as large industrial magnetomechanical sensors—pressductors [15] or accurate tensductors [16]—may be quantitatively described and optimized.

On the other hand, it should be stated that the proposed magnetoelastic effect description is limited only to isotropic materials, such as ferrites or non-grain oriented electrical steels. Moreover, the proposed model is limited to the initial area of the magnetization curve and to the initial range of mechanical stresses, where magnetic permeability as well as mechanical stress sensitivity can be considered nearly constant.
